# Low platelet count at admission has an adverse impact on outcome in patients with acute coronary syndromes: from the START Antiplatelet registry

**DOI:** 10.1038/s41598-024-64113-5

**Published:** 2024-06-24

**Authors:** Paolo Gresele, Giuseppe Guglielmini, Maurizio Del Pinto, Paolo Calabrò, Pasquale Pignatelli, Giuseppe Patti, Vittorio Pengo, Emilia Antonucci, Plinio Cirillo, Tiziana Fierro, Gualtiero Palareti, Rossella Marcucci, START Antiplatelet Registry Group, C. Riccini, A. Cesaro, F. Gragnano, D. Menichelli, D. Pastori, I. Cavallari, G. Denas, G. Zoppellaro, L. Di Serafino, G. De Rosa, G. Grossi, C. Piazzai

**Affiliations:** 1 Department of Medicine and Surgery, Section of Internal and Cardiovascular Medicine, University of Perugia, Strada Vicinale Via Delle Corse, S. Andrea Delle Fratte, 06132 Perugia, Italy; 2 Division of Cardiology, Perugia Hospital, Perugia, Italy; 3 Department of Translational Medical Sciences, University of Campania “Luigi Vanvitelli”, Naples, Italy; 4 Department of Clinical, Internistic, Anesthesiologic and Cardiovascular Sciences, Sapienza University of Rome, Rome, Italy; 5 Department of Translational Medicine, University of Eastern Piedmont, Maggiore della Carità Hospital, Novara, Italy; 6 Department of Cardiac, Thoracic, Vascular Sciences and Public Health, University of Padova, Padua, Italy; 7 Arianna Anticoagulazione Foundation, Bologna, Italy; 8 Division of Cardiology, Department of Advanced Biomedical Sciences, “Federico II” University, Naples, Italy; 9 Department of Experimental and Clinical Medicine, University of Florence, Florence, Italy

**Keywords:** Acute coronary syndromes, Platelets

## Abstract

Some previous observations suggest that a low platelet count is associated with an increased risk of adverse outcomes in patients with acute coronary syndromes (ACS). However, most of the data come from post-hoc analyses of randomized controlled trials and from studies including thrombocytopenia developed during hospital stay. Our aim was to assess the impact of low platelet count at admission on cardiovascular outcomes and treatment approach in patients hospitalized for ACS in a current real-life setting in Italy. Patients admitted to Italian coronary care units for ACS were enrolled in the START-ANTIPLATELET registry. Baseline clinical characteristics and treatment at discharge were recorded. Patients were followed-up at 6 months, 1 year and yearly thereafter. Low platelet count was defined as a count at admission < 150 > 100 k/µl or < 100 k/µL. Among 1894 enrolled patients, 157 (8.3%) had a platelet count < 150 > 100 k/µl and 30 (1.6%) < 100 k/µl. The median follow-up was 12.3 months (0.4–50.1). patients with low platelets were older (72 ± 10.4 vs 66 ± 12.4 years, p = 0.006), more frequently males (82.9 vs 72.1%, p = 0.001), hypertensive (90.0% vs 70.4%, p = 0.03), with non-valvular atrial fibrillation (NVAF) (17.1 vs 8.6%, p = 0.02), and peripheral arterial disease (11.5 vs 6.2% p = 0.01) and/or had a previous myocardial infarction (40 vs 18.7%, p = 0.008) and/or a PCI (14.6 vs 7.8%, p = 0.001) than patients with normal platelets. A slightly, but significantly, lower percentage of thrombocytopenic patients were treated with primary PCI (78.1 vs 84.4%, p = 0.04) and they were more frequently discharged on aspirin plus clopidogrel rather than aspirin plus newer P2Y_12_ antagonists (51.9 vs 65.4%, p = 0.01). MACE-free survival was significantly shorter in thrombocytopenic patients compared to patients with normal platelets (< 150 > 100 k/µl: 37.6 vs 41.8 months, p = 0.002; HR = 2.7, 95% CIs 1.4–5.2; < 100 k/µl: 31.7 vs 41.8 months, p = 0.01; HR = 6.5, 95% CIs 1.5–29.1). At multivariate analysis, low platelet count, age at enrollment, low glomerular filtration rate, low ejection fraction, a previous ischemic stroke and NVAF were independent predictors of MACE. A low platelet count at admission identifies a subgroup of ACS patients with a significantly increased risk of MACE and these patients should be managed with special care to prevent excess adverse outcomes.

## Introduction

Platelets play a crucial role in acute coronary syndromes (ACS) and several studies have consistently shown that an enhanced platelet count in patients undergoing an acute coronary event increases the relative risk of death and major adverse cardiovascular events (MACE), both in the short and in the long term^1–3^. Much less clear is the prognostic impact of a reduced platelet count in ACS patients. Thrombocytopenia is conventionally considered a risk factor for bleeding in patients presenting with an ACS treated with antithrombotic therapies^4,5^.

Thrombocytopenia develops during hospital stay in a significant fraction of ACS patients, either as a side effect of antithrombotic treatments or due to comorbidities, and its prognostic significance has been assessed in previous studies^6–8^. In a subanalysis of the PURSUIT trial, evaluating eptifibatide vs placebo in 10,948 patients with non-ST elevation ACS, thrombocytopenia developed in 70% of enrolled patients and they were more than twice as likely to experience moderate/severe bleeding than patients not developing thrombocytopenia^6^. In a cumulative analysis of 1001 patients enrolled in the TAMI and the Urokinase Trials, thrombocytopenia developed during the hospital stay in 16.4% of patients and these had significantly more hemorrhages than patients without thrombocytopenia^7^. In a post hoc analysis of the OASIS-2 Study, a trial which randomized 10,141 NSTE-ACS patients to i.v. unfractionated heparin or hirudin, patients who developed thrombocytopenia during drug infusion had a more than eight-fold increased incidence of major bleeding^8^.

On the other hand, several large epidemiological studies in the general population have shown that a reduced platelet count is independently associated with increased mortality, including cardiovascular mortality, suggesting that a low platelet count may represent a negative prognostic index among adult subjects^9–11^.

However, few studies have assessed the impact of a reduced platelet count at hospital admission in ACS patients on outcomes at follow-up and on antithrombotic treatment decisions.

The aim of our study was to evaluate whether a reduced platelet count at hospital admission has an impact on major adverse outcomes and antithrombotic treatment approach in patients with ACS in a current real-life setting in Italy.

## Results

### Baseline characteristics

Overall, 1894 patients were enrolled, 1707 (90.1%) had a normal platelet count (> 150 k/µl), 157 (8.3%) a platelet count < 150 > 100 k/µl, 18.4% of whom were females, and 30 (1.6%) < 100 k/µl, 16.7% of whom were females (Table 1). Patients with very low platelet count (i.e. < 50 k/µl) were 6 (0.3%) of the total population, 2 (33%) were females.

**Table 1:** Baseline clinical characteristics of the three ACS patient subpopulations.

	Normal count (1707, 90.1%)	Platelet < 150 > 100 k/µl (157, 8.3%)	Platelets < 100 k/µl (30, 1.6%)
Age, years (mean. sd)	65.6 (12.5)	*70.8 (10.8)	72 (10.4)
Males	1230 (72.1%)	*128 (81.5%)	25 (83.3%)
Hypertension	1200 (70.3%)	118 (76.6%)	*27 (90.0%)
Hypercholesterolemia	942 (55.2%)	86 (55.8%)	19 (63.3%)
Diabetes	449 (26.3%)	43 (27.9%)	12 (40.0%)
Obesity	371 (21.7%)	32 (20.8%)	*2 (6.7%)
Smoke	847 (49.6%)	70 (45.5%)	13 (43.3%)
NV AF	117 (6.9%)	*22 (14.3%)	4 (13.3%)
Valvular AF	22 (1.3%)	3 (1.9%)	1 (3.3%)
CVD familiarity	489 (28.6%)	38 (24.7%)	8 (26.7%)
Previous stroke/TIA	98 (5.7%)	13 (8.6%)	4 (13.3%)
Previous MI	318 (18.6%)	*48 (31.2%)	*12 (40.0%)
PAD	105 (6.2%)	*21 (11.2%)	*7 (25.0%)
Previous PCI	343 (20.1%)	*51 (33.1%)	10 (33.3%)
Valve prosthesis	12 (0.7%)	3 (1.9%)	1 (3.3%)
Previous major hemorrage	29 (1.7%)	5 (3.2%)	1 (3.3%)
Venous thromboembolism	15 (0.9%)	1 (0.6%)	1 (3.3%)
Pulmonary hypertension	4 (0.2%)	0 (0%)	0 (0%)
Ventricular thrombosis	11 (0.6%)	0 (0%)	0 (0%)
Ejection fraction % (mean. sd)	48.9 ± 10.9	49.4 ± 13.7	*43.6 (14.5)
Heart rate (bpm) (mean. sd)	70.7 ± 11.2	*74.0 ± 12.6	72.3 (11.2)

*AF* atrial fibrillation, *CVD* cardiovascular disease, *MI* myocardial infarction, *NV AF* non-valvular atrial fibrillation, *PAD* peripheral artery disease, *PCI* percutaneous coronary intervention, *TIA* transient ischemic attack.

*p < 0.05 vs normal count.

Patients with a low platelet count were older and more frequently males. Moreover, they had a higher prevalence of hypertension, had more often NVAF (13.9% vs 6.9%, p = 0.001) and peripheral arterial disease PAD (11.5 vs 6.2%, p = 0.01) than patients with a normal platelet count, and had suffered more frequently a previous MI and/or had undergone a previous PCI.

Concerning major cardiovascular risk factors (e.g. hypercholesterolemia, diabetes, smoke, family history of CV-disease, venous thromboembolism, valvular atrial fibrillation, pulmonary hypertension, ventricular thrombosis) these were similar in the two groups (Table 1).

At hospitalization, both groups of thrombocytopenic patients had lower hematocrit and hemoglobin levels than patients with normal platelet count, and also lower GFR (Table 2).

**Table 2:** Baseline laboratory values of the three ACS patient populations.

	Normal count (1707, 90.1%)	Platelet < 150 > 100 k/µl (157, 8.3%)	Platelets < 100 k/µl (30, 1.6%)
Hb (g/dl)	13.7 ± 1.9	*13.1 ± 2.0	*11.9 ± 2.0
Hct (%)	40.9 ± 5.5	*39.1 ± 5.9	*36.4 ± 5.6
Creatinine (mg)	1.1 ± 0.2	1.2 ± 0.1	1.3 ± 0.2
GFR (ml/min)	86.5 ± 41.2	*75.0 ± 33.2	*66.6 ± 30.5
ALT (U/l)	37.1 ± 28.9	40.2 ± 35.9	34.9 ± 25.7
AST (U/l)	61.1 ± 76.9	58.2 ± 78.2	61.2 ± 86.8
HbA1c (%)	6.9 ± 1.3	6.6 ± 0.3	6.4 ± 0.8

Data are means ± S.D.

*ALT* alanine amino transferase, *AST* aspartate transaminase, *GFR* glomerular filtration rate, *Hb* hemoglobin, *HbA1c* glycated hemoglobin, *Hct* hematocrit.

*p < 0.05 vs normal count.

No differences were found in terms of clinical presentation at hospital admission. Moreover, no significant difference was found regarding the type of therapeutic intervention adopted for ACS at the time of hospitalization, although a significantly lower fraction of thrombocytopenic patients underwent primary PCI compared with patients with a normal platelet count (Table 3).

**Table 3:** Clinical presentation and interventions adopted.

		Normal count (1707, 90.1%)	Platelet < 150 > 100 k/µl (157, 8.3%)	Platelets < 100 k/µl (30, 1.6%)
Clinical presentation	NSTEMI, n (%)	609 (35.7)	69 (43.9)	11 (36.7)
	STEMI, n (%)	882 (51.7)	59 (37.6)	13 (43.3)
	UA, n (%)	216 (12.7)	29 (18.5)	6 (20.0)
Intervention	CABG, n (%)	57 (3.3)	10 (6.7)	2 (3.4)
	Multivessel, n (%)	28 (1.6)	2 (16.7)	0 (0)
	Primary PCI, n (%)	1440 (84.3)	*119 (75.8)	25 (83.3)
	PCI + STENT, n (%)	1373 (80.4)	*113 (94.9)	23 (92.0)
	BMS, n (%)	40 (2.3)	4 (3.5)	2 (8.7)
	DES, n (%)	1333 (78.1)	*109 (96.5)	21 (91.3)
	Medical Therapy, n (%)	226 (13.2)	29 (19.3)	6 (20.0)
	DAPT, n (%)	143 (63.3)	15 (51.7)	4 (66.7)
	SAPT, n (%)	28 (12.4)	6 (20.7)	0 (0.0)
	Other, n (%)	55 (24.3)	8 (27.6)	2 (33.3)

*BMS* bare metal stent, *CABG* coronary artery bypass graft, *DAPT* dual antiplatelet therapy, *DES* drug eluting stent, *NSTEMI* non-ST elevation myocardial infarction, *PCI* percutaneous coronary intervention, *SAPT* single antiplatelet therapy, *STEMI* ST elevation myocardial infarction, *UA* unstable angina.

Other: Adenosine/Heparin/Dobutamine; Adenosine/Venitrin/ReoPro; ASA/Apixaban; ASA/Coumadin; ASA/heparin/Captopril; ASA/heparin/nitrates; ASA/Heparin; ASA/LMWH/Rytmonorm; Beta Blockers; Bivalirudin/dobutamine; Clopidogrel/Heparin; Fibrinolysis; fondaparinux; Heparin/Atropin/Effortil; Heparin/Ticagrelor; Indomethacin/Acenocumarol; Ivabradine/nitrates; Nitrates/LMWH/Zyllt; Nitrates; ReoPro/Heparin; ReoPro; Tenecteplase.

*p < 0.05 vs normal count.

### Medical therapy at discharge

Both groups of patients were discharged with similar treatments, except for a slightly but significantly higher prevalence of thrombocytopenic patients discharged on aspirin plus clopidogrel rather than on aspirin plus newer P2Y_12_ antagonists (Table 4).

**Table 4:** Treatment at hospital discharge.

		Normal count (1707, 90.1%)	Platelet < 150 > 100 k/µl (157, 8.3%)	Platelets < 100 k/µl (30, 1.6%)
Treatment at discharge	DAPT (n,% of the total population)	1581 (92.6%)	*134 (85.3%)	27 (90.0%)
	ASA-CLOP (n,%)	464 (29.3%)	*52 (38.8%)	*19 (70.3%)
	ASA-PRAS (n,%)	178 (11.2%)	13 (9.7%)	1 (3.7%)
	ASA-TICA (n,%)	939 (59.4%)	69 (51.5%)	7 (25.9%)
	SAPT (N,%)	113 (6.6%)	23 (14.6%)	3 (10.0%)
	OAC (overall) (n,%)	133 (7.8%)	*22 (14.2%)	6 (20.0%)
	OAC (n,%)	2 (1.5%)	2 (9.1%)	0 (0%)
	OAC + SAPT (n,%)	24 (18.0%)	6 (27.3%)	0 (0%)
	OAC + DAPT (n,%)	107 (80.4%)	14 (63.6%)	6 (20.0%)
	Statins (n,%)	1640 (96.1%)	154 (98.1%)	28 (93.3%)
	ACE-I/ARB (n,%)	1140 (66.8%)	100 (63.7%)	12 (40.0%)
	Beta block (n,%)	1211 (70.9%)	109 (72.7%)	18 (60%)
	CA (n,%)	70 (8.2%)	7 (4.5%)	0 (0%)
	Nitrates (n,%)	138 (8.1%)	26 (16.5%)	2 (6.7%)
	Diuretics (n,%)	431 (25.2%)	50 (31.8%)	14 (46.7%)
	PPI (n,%)	1648 (96.5%)	154 (98.1%)	27 (90.0%)

*ACE-I/ARB* ACE inhibitors/angiotensin receptor blockers, *ASA-CLOP* aspirin plus clopidogrel, *ASA-PRAS* aspirin plus prasugrel, *ASA-TICA* aspirin plus ticagrelor, *BETA BLOCK* beta blockers, *CA* calcium antagonists, *DAPT* dual antiplatelet therapy, *OAC* oral anticoagulants, *PPI* proton pump inhibitors, *SAPT* single antiplatelet therapy.

*p < 0.05 vs normal platelet count (≥ 150 k/µl).

Patients with atrial fibrillation, both valvular and non-valvular were 161 of whom 59% were discharged on vitamin K inhibitors, 22.4% were on DOAC (Dabigatran 8.7%, Apixaban 7.5%, Rivaroxaban 6.2%).

DAPT was prescribed for 12 months. 93.9% of the patients cohort maintained dual therapy for the duration prescribed, 5.9% received an extension of therapy upto 24 months, and 0.2% for further 12 months.

### Clinical outcomes

Median follow-up time was 12.3 months (0.4–50.1 months). MACE occurred in 117 patients of the total patient population (6.3%), of whom 15 (11 cardiovascular death, 3 MI, 1 thrombotic complication) in patients with a platelet count < 150 > 100 k/µl (10.7%) and 5 (4 cardiovascular death and 1 MI) in patients with a platelet count < 100 k/µl (16.7%) (Table 5). None of the patients with very low platelet count (i.e. < 50 k/µl) suffered a MACE at follow-up. Excluding those patients from the analysis did not change the results.

**Table 5:** Cardiovascular complications at follow-up.

	Normal count (1707, 90.1%)	Platelet < 150 > 100 k/µl (157, 8.3%)	Platelets < 100 k/µl (30, 1.6%)
CV death	39 (40.2%)	*11 (73.3%)	*4 (80.0%)
AMI	21 (21.6%)	3 (20.0%)	1 (20.0%)
Stroke	12 (12.4%)	0 (0%)	0 (0%)
TIA	1 (1.0%)	0 (0%)	0 (0%)
TVR	22 (22.7%)	0 (0%)	0 (0%)
Peripheral embolism	2 (2.0%)	1 (6.7%)	0 (0%)
Total (% of total population)	97 (5.7%)	*15 (9.5%)	*5 (16.7%)

*AMI* acute myocardial infarction, *CV Death* cardiovascular death, *TIA* transient ischemic attack, *TVR* target vessel revascularization.

*p < 0.05 vs normal count (≥ 150 k/µl).

**Figure 1:**
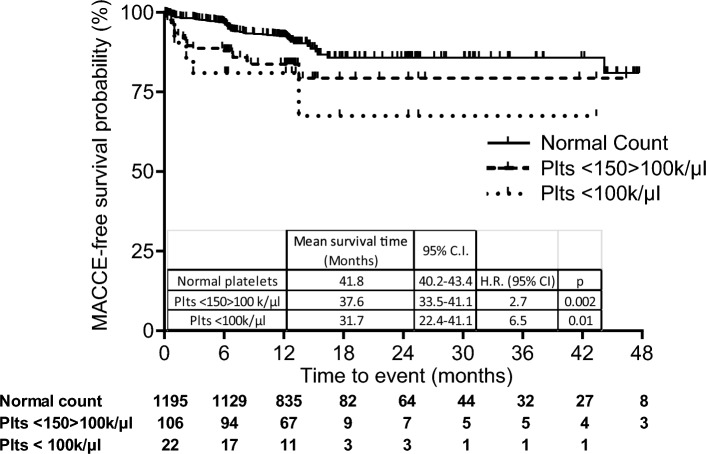
MACE-free survival probability in ACS patients with a normal platelet count, mild (< 150 > 100 k/µl) or moderate (< 100 k/µl) thrombocytopenia. Number of patients included in the follow-up are reported under the figure. Patients lost at follow-up were 0.6% in the mild thrombocytopenic group and 0.9% in the moderate thrombocytopenic group.

MACE-free survival of patients with thrombocytopenia was significantly shorter compared to patients with a normal platelet count (platelet count < 150 > 100 k/µl: 37.6 months, 95% CIs 33.5–41.1 vs 41.8 months, 40.2–43.4, HR: 2.7; 1.4–5.2, p = 0.002, platelet count < 100 k/µl: 31.7 months, 95% CI 22.4–41.1, HR: 6.5, 1.5–29.1, p = 0.01) (Fig. 1). Interestingly, MACE incidence in patients with platelet count < 150 k/µl was inversely correlated with the increase of platelet count (r^2^ = 0.751, p = 0.025) (Supplemental Fig. 1).

Bleeding events were registered in 120 patients (6.4%), and were classified according with the GUSTO criteria as severe 0.9%, moderate 1.1% and mild 4.4%. In patients with platelet count < 150 > 100 k/µl hemorrhagic events were registered in a total of 5.8% of the population and were severe in 0.5%, moderate in 0.5% and mild in 4.8%, while in patients with platelet < 100 k/µl they were registered in 2.7% of the population and were all moderate.

**Figure 2:**
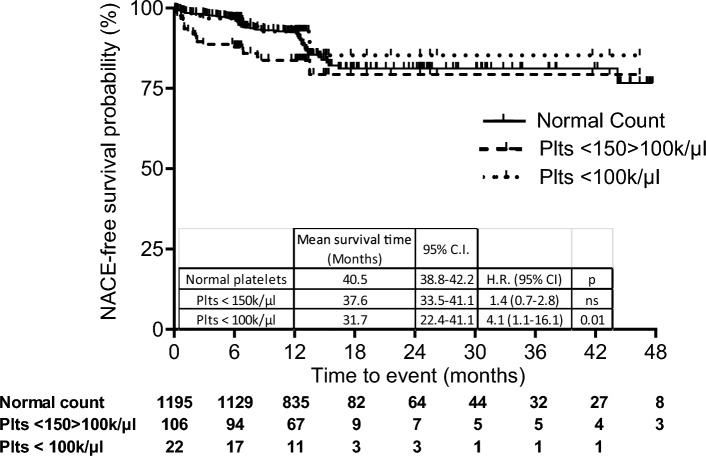
NACE-free survival curves in ACS patients with a normal platelet count, mild (< 150 > 100 k/µl) or moderate thrombocytopenia (platelets < 100 k/µl). Number of patients included in the follow-up are reported under the figure.

Patients with platelet < 150 > 100 k/µl had a significantly shorter NACE-free survival time than patients with normal platelets [37.6 months, 33.5–41.1, vs 40.5 months, 38.8–22.2; HR = 2.1; 1.2–3.8, p = 001]; and even more so patients with a platelet count < 100 k/μl (31.7 months, 22.4–41.1, HR = 4.0, 1.0–15.5, p = 0.05) (Fig. 2).

**Figure 3:**
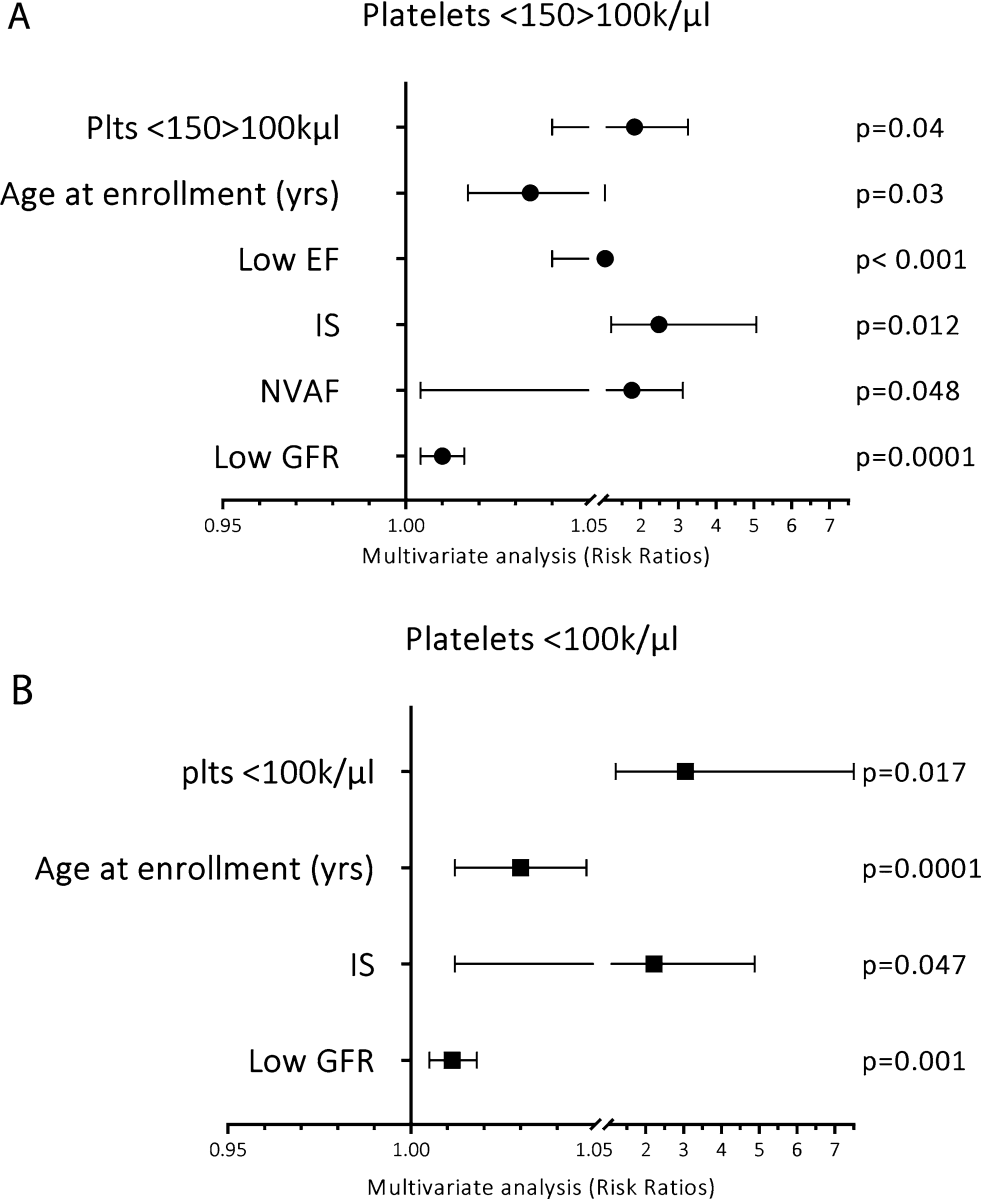
Multivariate COX regression analysis showing the variables independently associated with MACE in patients with a platelet count < 150 > 100 k/µl (**A**) and < 100 k/µl (**B**). Data show hazard ratios and 95% CIs. *EF* ejection fraction, *IS* ischemic stroke, *NVAF* non-valvular atrial fibrillation, *GFR* glomerular filtration rate.

Multivariate analysis showed that low platelet count (< 150 k/µl) is strongly associated with MACE at follow-up with an adjusted OR = 1.96 (1.2–3.3), p = 0.008. Furthermore, platelet count < 150 > 100 k/µl was an independent predictor of MACE (OR = 1.84, 1.04–3.25, p = 0.04), and even more a platelet count < 100 k/µl (OR = 3.05, 1.2–7.5, p = 0.017). Moreover, both among patients with a platelet count < 150 > 100 k/µl and patients with a platelet count < 100 k/µl, higher age at enrollment was predictive of MACE at follow-up (OR = 1.034, 1.017–1.052, p = 0.0001; and 1.03, 1.012–1.048, p = 0.0001) (Fig. 3).

Finally, in patients with a platelet count < 150 > 100 k/µl low GFR (OR = 1.1, 1.0–1.01, p < 0.0001), NVAF (OR = 1.8, 1.0–3.1, p = 0.048), low EF (OR = 1.1, 1.04–1.08, p < 0.0001), and a previous IS (OR = 2.5, 1.2–5.1, p = 0.01) were predictive of MACE (Fig. 3A), while in patients with platelet count < 100 k/µl, low GFR (OR = 1.01, 1.005–1.02, p = 0.001) and a previous IS (OR = 2.2, 1.01–4.9, p = 0.047) were predictive of MACE (Fig. 3B).

In the overall population, both previous MI and the combination of previous MI plus previous PCI were independently associated to MACE with HR = 1.6 (1.1–2.5), p = 0.02, and 1.5 (1.01–2.3), 9 = 0.04, respectively, confirming literature data.

## Discussion

Our data, derived from a real-life registry of patients hospitalized for an ACS in Italy, show that the presence of a reduced platelet count at admission is associated with a worse outcome, with a higher incidence of MACE and NACE, an effect apparently correlated with the degree of thrombocytopenia.

In our ACS population, a mild thrombocytopenia at admission was found in 8.3% of the patients and a moderate thrombocytopenia in 1.6%. Previous studies exploring the impact of the platelet count at admission on outcomes in patients with ACS reported a prevalence of thrombocytopenia ranging from 5.7 to 21.3%^2,20–24^, in line with our data, showing that thrombocytopenia is not an infrequent finding in patients hospitalized for an ACS.

While emphasis on the risks associated with thrombocytopenia in ACS patients was previously placed on hemorrhagic complications^3–6^, our study shows that the main adverse outcome associated with thrombocytopenia is MACE.

Low platelet count identifies a group of ACS patients with increased cardiovascular risk^2,25^, and mild degree of thrombocytopenia at hospital admission has been associated with in-hospital cardiovascular complications and with short- and long-term mortality^21^, in particular in patients undergoing PCI^20^. In a subanalysis of the HORIZONS-AMI Study, baseline thrombocytopenia was found in 4.2% of patients and was associated with a significantly increased MACE at 30 days, with 9.6% of patients experiencing a cardiac event vs 5.2% in those without thrombocytopenia (p < 0.05)^25^. In a retrospective study on 9531 patients admitted to the West China Hospital for a PCI, 9.8% with thrombocytopenia at admission had a significantly higher incidence of primary outcome and target vessel revascularization^26^. In an analysis of patients discharged from Mayo Clinic Arizona, out of 536 patients, 72 (13%) had thrombocytopenia and over a follow-up of 1.1 years they had higher all-cause mortality but not major bleeding events^27^. A metanalysis, including 8 studies and almost 40,000 ACS patients, showed an U-shaped curve for the association of platelet count at admission with MACE, with both reduced and increased platelet counts associating with enhanced MACE^1^.

Thrombocytopenic patients in our cohort had a relatively higher cardiovascular risk profile compared with patients with a normal count. Thrombocytopenic ACS patients, both mild and moderate, were older, more frequently hypertensive and with NVAF or PAD, and had more often suffered a previous MI than ACS patients with a normal platelet count. They also had lower hemoglobin, hematocrit and impaired GFR. The higher risk profile of our cohort of thrombocytopenic patients with ACS led to a worse prognosis, despite clinical presentation was similar to that of patients with a normal platelet count. Moreover, MACE incidence in thrombocytopenic patients (i.e. platelet count < 150 k/µl) was inversely correlated with the platelet count. Indeed, cardiovascular mortality was higher in thrombocytopenic patients (79% and 80% vs 40.2%, p = 0.025) and MACE-free survival time was significantly shorter in both mild and moderate thrombocytopenic patients, and age at enrollment, previous ischemic stroke, and low GFR predicted MACE at multiple regression analysis in both groups of thrombocytopenic patients. Moreover, in mild but not in moderate thrombocytopenic patients, MACE were also independently associated with a lower ejection fraction and with NVAF, but the difference found between mild and moderate thrombocytopenia can be due to the low number of moderate thrombocytopenic patients in our cohort.

In terms of therapeutic implications of our findings, it is of note that in patients with a recent ACS, with or without coronary revascularization, a low platelet count is considered a major of bleeding risk criterion by the high bleeding risk (HBR) definition^28^. In these HBR patients the 2023 ESC Guidelines for the management of ACS^29^ consider the possibility of shortening dual antiplatelet therapy or de-escalating from potent P2Y_12_ inhibitors (prasugrel or ticagrelor) to clopidogrel. Evidence from our study points out that patients with a low platelet count have increased MACE risk especially in the first 3–6 months after ACS thus, our data suggest that a default strategy of shortening DAPT or de-escalating from potent P2Y_12_inhibitors to clopidogrel in thrombocytopenic patients should be carefully weighted with the thrombotic risk, especially in those patients with complex PCI (eg. left main bifurcation stenting), while mono-therapy with ticagrelor after 1–3 months of DAPT could be an option in patients with a high bleeding risk^30,31^.

## Conclusions

In conclusion, low platelet count identifies a subgroup of ACS patients at significantly increased risk of adverse outcomes. Patients admitted to CCU for ACS with a low platelet count should be treated with special care to prevent excess adverse outcomes. Older patients with ACS and low platelet count are currently treated in a more conservative manner, with less frequent revascularization and lower intensity antiplatelet regimen^29^. Our results suggest however that these patients have a high CV-risk profile (previous AMI in 40% in low platelet count vs 18% normal count) and a higher incidence of MACE. Therefore, in this complex patient population a careful assessment of the individual bleeding and thrombotic risk is highly warranted. It might be suggested that in this specific subset of patients a shorter DAPT duration together with a closer follow-up could also be considered. A strategy of DAPT with Aspirin and Ticagrelor for only one month withholding aspirin subsequently, as used in the Global Leader trial^32^ and suggested by ESC 2023 Guidelines^29^, might be envisaged.

Moreover, a stricter glycemic control, lipid-lowering treatment to attain goal LDL-C levels and use of proton pump inhibitors should be adopted^31^.

## Methods

START-ANTIPLATELET, a branch of the START registry (Survey on anTicoagulated pAtients RegisTer) (NCT02219984), is a prospective, real-life multi-center observational registry started on January 2014 and involving 8 Italian centers, which evaluates current treatment and outcomes of patients hospitalized for ACS in Italy^12–16^. The registry was investigators-driven, non-sponsored and was approved by the Ethic Committee of each participating institution (Campus Bio-Medico University of Rome; Monaldi Hospital and "Luigi Vanvitelli" University of Campania; "Federico II" University of Naples; University of Perugia; University Hospital of Padua; La Sapienza University of Rome; University of Florence).

Consecutive patients presenting with an ACS [STE-myocardial infarction (STEMI), non-STE-ACS, and unstable angina (UA)] who either underwent revascularization or were treated with medical therapy, were enrolled. To exclude selection bias, two specific and fixed working days in the week were chosen for patient enrollment at each site and all patients admitted on those days were included in the registry, independently from clinical presentation. Patients undergoing elective percutaneous coronary interventions (PCI) were excluded. The only additional exclusion criteria were hematologic disease or tumor radiotherapy and chemotherapy and the inability or unwillingness to give written informed consent for enrollment in the study and the simultaneous participation in another research study. An informed consent was obtained from all subjects and/or their legal guardian(s).

Standard of care for PCI and related management were adopted at the discretion of the treating physicians. The accurate collection of baseline demographics, clinical characteristics, laboratory data and treatments applied was the responsibility of the participating investigators.

Clinical data were registered at hospital admission (baseline visit) and follow-up reassessments were made at 6 months, 1 year, and yearly thereafter either through outpatient visits or, whenever not possible, telephonic interviews.

All methods were performed in accordance with the relevant guidelines and regulations of the Ethic Committee of each participating institution.

The primary endpoint was MACE at one year, defined as a composite of cardiovascular death, non-fatal myocardial infarction (MI), transient ischemic attack (TIA), ischemic stroke (IS), target vessel revascularization (TVR) and major arterial ischemic events. The co-primary endpoint was one-year net adverse cardiovascular events (NACE), including MACE and major bleeding. Secondary endpoints included individual components of the primary endpoint and clinically significant bleeding as well as MACE plus all-cause death^17^. Bleeding events during or after hospitalization were classified according to the GUSTO criteria as mild (not consistent with the moderate or severe bleeding criteria), moderate (bleeding requiring blood transfusions but not causing hemodynamic impairment), or severe (intracranial hemorrhage or bleeding causing hemodynamic impairment)^18^.

Hemorrhagic events and post discharge MACE were independently validated by the participating investigators through a review of medical records.

For the diagnosis of NSTE-ACS or STEMI, ischemic symptoms and at least 1 of the following were required: new ST-T wave variations, new left bundle-branch block, pathological Q-waves on electrocardiogram or regional cardiac wall movement anomalies at imaging. Ischemic stroke was defined as loss of neurological function caused by an ischemic event with symptoms lasting ≥ 24 h or causing death. Unplanned coronary revascularization included any coronary artery bypass graft (CABG) surgery or repeated PCI carried out after the index PCI. Scheduled revascularization procedures at the time of the index PCI and occurring within 60 days were not considered target vessel revascularization events, unless a recurrent ischemic episode determined the timing of the new procedure^12^.

Low platelet count was defined as a count at admission < 150 > 100 k/µl an a further severity category was defined by a count of < 100 k/µl.

### Statistical analysis

Categorical variables are reported as numbers and percentages and were compared using the chi-square test. Continuous variables are reported as means and standard deviations and were compared by the t-test for normally distributed data (as assessed by Kolmogorov–Smirnov test), or by the Mann–Whitney U-test for not-normally distributed data. Clinical event rates were analyzed with the Kaplan–Meier method and compared using the Breslow statistics. Multivariable analysis was performed by logistic regression following published guidelines^19^ for the variables with p < 0.1, which were: for patients with platelet count < 150 > 100 k/µl: sex, age at enrollment, hypertension, previous MI, previous PCI, previous ischemic stroke, NVAF and peripheral arterial disease (PAD); and for patients with platelet count < 100 k/µl: age at enrollment, hypertension, obesity (BMI > 30 kg/m^2^), previous acute myocardial infarction (AMI) and PAD, hemoglobin and hematocrit levels and glomerular filtration rates. Variables were removed from the final model when p > 0.1 and expressed as odds ratios and 95% C.I. All data were analyzed using the IBM SPSS 25 software, and a two-tailed p-value < 0.05 was considered as statistically significant.

### Supplementary Information

Supplementary Figure 1.

## Data Availability

The data underlying this article will be shared on reasonable request to the corresponding author.

## References

[1] Wu Y, Wu H, Mueller C, Gibson CM, Murphy S, Shi Y, Xu G, Yang J (2012). Baseline platelet count and clinical outcome in acute coronary syndrome. Circ. J..

[2] Song PS, Ahn KT, Jeong JO, Jeon KH, Song YB, Gwon HC, Rha SW, Jeong MH, Seong IW (2020). Association of baseline platelet count with all-cause mortality after acute myocardial infarction. Eur. Heart J. Acute Cardiovasc. Care..

[3] Galimzhanov A, Sabitov Y, Tenekecioglu E, Tun HN, Alasnag M, Mamas MA (2022). Baseline platelet count in percutaneous coronary intervention: A dose-response meta-analysis. Heart..

[4] Discepola V, Schnitzer ME, Jolicoeur EM, Rousseau G, Lordkipanidzé M (2019). Clinical importance of thrombocytopenia in patients with acute coronary syndromes: A systematic review and meta-analysis. Platelets..

[5] Martens KL, Dekker SE, Crowe M, DeLoughery TG, Shatzel JJ (2022). Challenging clinical scenarios for therapeutic anticoagulation: A practical approach. Thromb. Res..

[6] McClure MW, Berkowitz SD, Sparapani R, Tuttle R, Kleiman NS, Berdan LG, Lincoff AM, Deckers J, Diaz R, Karsch KR, Gretler D, Kitt M, Simoons M, Topol EJ, Califf RM, Harrington RA (1999). Clinical significance of thrombocytopenia during a nonSTelevation acute coronary syndrome: The platelet glycoprotein IIb/IIIa in unstable angina: Receptor suppression using integrilin therapy (PURSUIT) trial experience. Circulation..

[7] Harrington RA, Sane DC, Califf RM, Sigmon KN, Abbottsmith CW, Candela RJ, Lee KL, Topol EJ (1994). Clinical importance of thrombocytopenia occurring in the hospital phase after administration of thrombolytic therapy for acute myocardial infarction: The Thrombolysis and Angioplasty in Myocardial Infarction Study Group. J. Am. Coll. Cardiol..

[8] Eikelboom JW, Anand SS, Mehta SR, Weitz JI, Yi C, Yusuf S (2001). Prognostic significance of thrombocytopenia during hirudin and heparin therapy in acute coronary syndrome without ST elevation: Organization to Assess Strategies for Ischemic Syndromes (OASIS-2) study. Circulation..

[9] Vinholt PJ, Hvas AM, Frederiksen H, Bathum L, Jørgensen MK, Nybo M (2016). Platelet count is associated with cardiovascular disease, cancer and mortality: A population based cohort study. Thromb. Res..

[10] Kabat GC, Kim MY, Verma AK, Manson JE, Lin J, Lessin L, Wassertheil-Smoller S, Rohan TE (2017). Platelet count and total and cause-specific mortality in the Women's Health Initiative. Ann. Epidemiol..

[11] Bonaccio M, Di Castelnuovo A, Costanzo S, De Curtis A, Donati MB, Cerletti C, de Gaetano G, Iacoviello L (2018). Age- and sex-based ranges of platelet count and cause-specific mortality risk in an adult general population: prospective findings from the Moli-sani study. Platelets..

[12] Marcucci R, Patti G, Calabrò P (2019). Antiplatelet treatment in acute coronary syndrome patients: Real-world data from the START-Antiplatelet Italian Registry. PLoS One..

[13] Calabrò P, Gragnano F, Di Maio M, Patti G, Antonucci E, Cirillo P, Gresele P, Palareti G, Pengo V, Pignatelli P, Pennacchi M, Granatelli A, De Servi S, De Luca L, Marcucci R (2018). Epidemiology and management of patients with acute coronary syndromes in contemporary real-world practice: Evolving trends from the EYESHOT study to the START-ANTIPLATELET registry. Angiology..

[14] Cesaro A, Gragnano F, Calabrò P, Moscarella E, Santelli F, Fimiani F, Patti G, Cavallari I, Antonucci E, Cirillo P, Pignatelli P, Palareti G, Pelliccia F, Bossone E, Pengo V, Gresele P, Marcucci R (2021). Prevalence and clinical implications of eligibility criteria for prolonged dual antithrombotic therapy in patients with PEGASUS and COMPASS phenotypes: Insights from the START-ANTIPLATELET registry. Int. J. Cardiol..

[15] Calabrò P, Moscarella E, Gragnano F, Cesaro A, Pafundi PC, Patti G, Cavallari I, Antonucci E, Cirillo P, Pignatelli P, Palareti G, Sasso FC, Pengo V, Gresele P, Marcucci R, Conte M, Fimiani F, Di Serafino L, Del Pinto M, Denas G, Pastori D, Eleonora C, Fierro T (2019). Effect of body mass index on ischemic and bleeding events in patients presenting with acute coronary syndromes (from the START-ANTIPLATELET registry). Am. J. Cardiol..

[16] Gragnano F, Moscarella E, Calabrò P, Cesaro A, Pafundi PC, Ielasi A, Patti G, Cavallari I, Antonucci E, Cirillo P, Pignatelli P, Palareti G, Pelliccia F, Gaudio C, Sasso FC, Pengo V, Gresele P, Marcucci R (2021). Clopidogrel versus ticagrelor in high-bleeding risk patients presenting with acute coronary syndromes: Insights from the multicenter START-ANTIPLATELET registry. Intern. Emerg. Med..

[17] Gresele P, Guglielmini G, Del Pinto M, Calabrò P, Pignatelli P, Patti G, Pengo V, Antonucci E, Cirillo P, Fierro T, Palareti G, Marcucci R (2021). Peripheral arterial disease has a strong impact on cardiovascular outcome in patients with acute coronary syndromes: From the START Antiplatelet registry. Int. J. Cardiol..

[18] The GUSTO Investigators (1993). An international randomized trial comparing four thrombolytic strategies for acute myocardial infarction. N. Engl. J. Med..

[19] Vittinghoff E, McCulloch CE (2007). Relaxing the rule of ten events per variable in logistic and Cox regression. Am. J. Epidemiol..

[20] Overgaard CB, Ivanov J, Seidelin PH, Todorov M, Mackie K, Dzavík V (2008). Thrombocytopenia at baseline is a predictor of in hospital mortality in patients undergoing percutaneous coronary intervention. Am. Heart J..

[21] Sinkovič A, Majal M (2015). The impact of thrombocytopenia on outcome in patients with acute coronary syndromes: A single center retrospective study. Biomed. Res. Int..

[22] Yadav M, Généreux P, Giustino G, Madhavan MV, Brener SJ, Mintz G, Caixeta A, Xu K, Mehran R, Stone GW (2016). Effect of baseline thrombocytopenia on ischemic outcomes in patients with acute coronary syndromes who undergo percutaneous coronary intervention. Can. J. Cardiol..

[23] Małyszczak A, Łukawska A, Dyląg I, Lis W, Mysiak A, Kuliczkowski W (2020). Blood platelet count at hospital admission impacts long-term mortality in patients with acute coronary syndrome. Cardiology..

[24] Patti G, Di Martino G, Ricci F, Renda G, Gallina S, Hamrefors V, Melander O, Sutton R, Engström G, De Caterina R, Fedorowski A (2019). Platelet indices and risk of death and cardiovascular events: Results from a large population-based cohort study. Thromb. Haemost..

[25] Hakim DA, Dangas GD, Caixeta A, Nikolsky E, Lansky AJ, Moses JW, Claessen B, Sanidas E, White HD, Ohman EM, Manoukian SV, Fahy M, Mehran R, Stone GW (2011). Impact of baseline thrombocytopenia on the early and late outcomes after ST-elevation myocardial infarction treated with primary angioplasty: Analysis from the Harmonizing Outcomes with Revascularization and Stents in Acute Myocardial Infarction (HORIZONS-AMI) trial. Am. Heart J..

[26] Chen Z, Liu Z, Li N, Liu R, Wang M, Wang D, Li C, Li K, Luo F, He Y (2021). Impact of thrombocytopenia on in-hospital outcome in patients undergoing percutaneous coronary intervention. Cardiovasc. Ther..

[27] Chao CJ, Shanbhag A, Chiang CC, Girardo ME, Seri AR, Khalid MU, Rayfield C, O'Shea MP, Fatunde O, Fortuin FD (2021). Baseline thrombocytopenia in acute coronary syndrome: The lower, the worse. Int. J. Cardiol..

[28] Urban P, Mehran R, Colleran R (2019). Defining high bleeding risk in patients undergoing percutaneous coronary intervention: A consensus document from the academic research consortium for high bleeding risk. Circulation..

[29] Byrne RA, Rossello X, Coughlan JJ, Barbato E, Berry C, Chieffo A, Claeys MJ, Dan GA, Dweck MR, Galbraith M, Gilard M, Hinterbuchner L, Jankowska EA, Jüni P, Kimura T, Kunadian V, Leosdottir M, Lorusso R, Pedretti RFE, Rigopoulos AG, Rubini Gimenez M, Thiele H, Vranckx P, Wassmann S, Wenger NK, Ibanez B (2023). 2023 ESC Guidelines for the management of acute coronary syndromes. Eur. Heart J..

[30] Kim BK, Hong SJ, Cho YH, Yun KHo, Kim YH, Suh Y,  (2020). Effect of ticagrelor monotherapy vs ticagrelor with aspirin on major bleeding and cardiovascular events in patients with acute coronary syndrome: The TICO randomized clinical trial. JAMA.

[31] Guo H, Ye Z, Huang R (2021). Clinical outcomes of concomitant use of proton pump inhibitors and dual antiplatelet therapy: A systematic review and meta-analysis. Front. Pharmacol..

[32] Vranckx P, Valgimigli M, Jüni P, Hamm C, Steg PG, Heg D, van Es GA, McFadden EP, Onuma Y, van Meijeren C, Chichareon P, Benit E, Möllmann H, Janssens L, Ferrario M, Moschovitis A, Zurakowski A, Dominici M, Van Geuns RJ, Huber K, Slagboom T, Serruys PW, Windecker S (2018). Ticagrelor plus aspirin for 1 month, followed by ticagrelor monotherapy for 23 months vs aspirin plus clopidogrel or ticagrelor for 12 months, followed by aspirin monotherapy for 12 months after implantation of a drug-eluting stent: a multicentre, open-label, randomised superiority trial. Lancet..

