# Supplementary material for: Low platelet count at admission has an adverse impact on outcome in patients with acute coronary syndromes: from the START Antiplatelet registry
Source: Sci Rep. 2024 Jun 24;14:14516. doi: 10.1038/s41598-024-64113-5 (PMC11196263; doi:10.1038/s41598-024-64113-5)

Supplemental Material

Supplemental Figure 1

Correlation of platelet count at admission with MACE incidence in patients with platelet count <150k/µl. Data are presented as regression line and 95%CI of regression line. R^2= 0.751, p=0.025.

Supplemental Figure 1

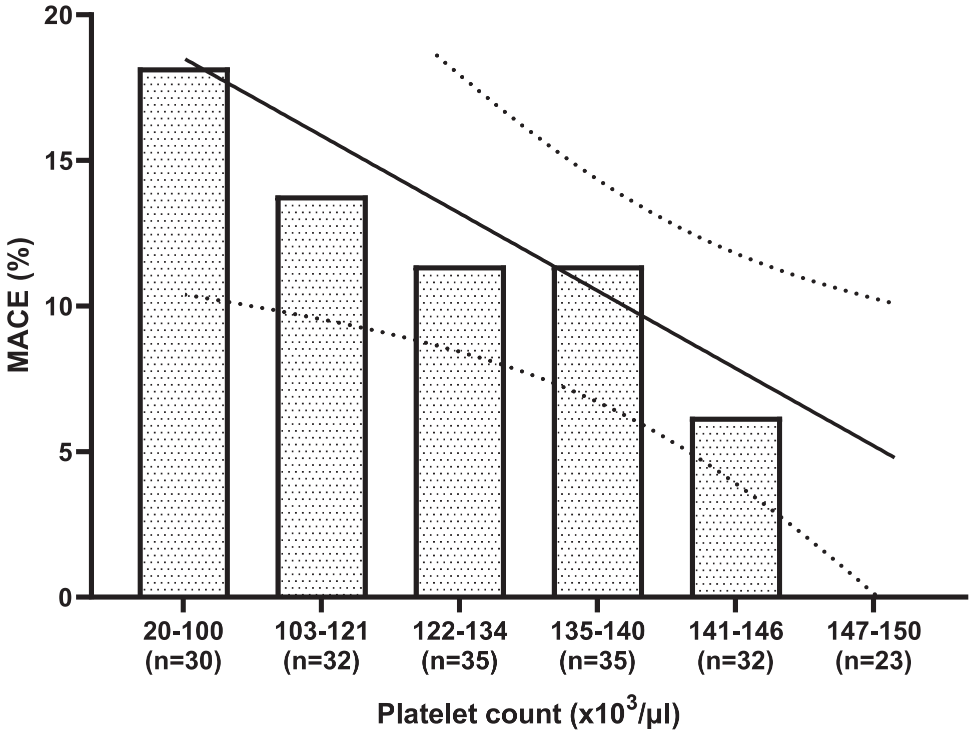

Supplement: Supplementary file 1 — Supplementary Figure 1. [file 41598_2024_64113_MOESM1_ESM.pdf]
